# Personality Predicts Social Dominance in Male Domestic Fowl

**DOI:** 10.1371/journal.pone.0103535

**Published:** 2014-07-29

**Authors:** Anna Favati, Olof Leimar, Hanne Løvlie

**Affiliations:** 1 Department of Zoology, Stockholm University, Stockholm, Sweden; 2 IFM Biology, Linköping University, Linköping, Sweden; Uppsala University, Sweden

## Abstract

Individuals in social species commonly form dominance relationships, where dominant individuals enjoy greater access to resources compared to subordinates. A range of factors such as sex, age, body size and prior experiences has to varying degrees been observed to affect the social status an individual obtains. Recent work on animal personality (i.e. consistent variation in behavioural responses of individuals) demonstrates that personality can co-vary with social status, suggesting that also behavioural variation can play an important role in establishment of status. We investigated whether personality could predict the outcome of duels between pairs of morphologically matched male domestic fowl (*Gallus gallus domesticus*), a species where individuals readily form social hierarchies. We found that males that more quickly explored a novel arena, or remained vigilant for a longer period following the playback of a warning call were more likely to obtain a dominant position. These traits were uncorrelated to each other and were also uncorrelated to aggression during the initial part of the dominance-determining duel. Our results indicate that several behavioural traits independently play a role in the establishment of social status, which in turn can have implications for the reproductive success of different personality types.

## Introduction

In social species, relationships often take the form of dominance hierarchies, commonly described as the repeated win of one individual over another [1]. Individuals that obtain a dominant position commonly enjoy increased access to resources, such as mating partners, which can result in a positive relationship between social status and reproductive success and thereby fitness [2]. Morphological traits and body size can contribute to the assessment of an antagonist [2]. Various other factors like kinship, prior dominance experience or residency, can further increase the probability of gaining a dominant position [3]–[5].

Behavioural predictors of dominance have received much less attention than morphological characters, even though dominant and subordinate individuals commonly differ quantitatively in behaviour. For example, dominant individuals are often more aggressive than subdominant individuals [6]–[8], but see [9]. Individuals of different social status may also differ in behavioural responses like boldness [10] and explorative tendencies [11]. In addition, variation among individuals in these behavioural traits is often consistent across time and/or context, and is used to describe an individual’s personality type [12].

Dominance and aggression are sometimes included in the concept of coping styles, a composite classification of behavioural profiles including description of individuals by their activity level, speed of exploration and boldness [13]. Individuals that have a higher general activity, quickly explore new environments and are more aggressive are said to have a ‘proactive’ coping style [13]–[15]. ‘Reactive’ individuals on the other hand, are characterised by being more passive, and less bold, explorative and aggressive [13]–[15]. The definition of coping styles assumes that these traits are positively correlated within individuals, which they sometimes [10], [11], [16], but not always, are [17], [18]. Variation in coping styles is at least partly explained by differences in stress hormone profiles, with proactive individuals having a more pronounced sympathetic stress activation (flight/fight), while reactive individuals often respond to a stressful situation by a higher parasympathetic stress activation (withdrawal reaction) [13]. Individual differences in coping with challenges experienced during behavioural assays may reflect variation in coping styles during the challenge of meeting a same-sexed conspecific in a social duel. Some studies have used animals selected for variation in proactivity or aggression, with the results indicating that proactive and aggressive individuals have higher chances of obtaining a high social status [19], [20]. However, studies of whether natural variation in personality can predict social status are rare. The few studies available show an overall trend that proactive individuals more often become dominant [11], [15], [21]–[23], but there are also examples of the opposite [18]. The potential for personality variation to predict social status is therefore still unclear.

We have tested the predictive power of behavioural responses in a non-social context for the establishment of social status in male domestic fowl (*Gallus gallus domesticus*). The fowl is a group-living species that readily forms social hierarchies where individuals occupy clear social roles [24]. Despite the long history of studies on social hierarchies in the domestic fowl, the traits determining status are not fully understood, especially not in males. Ornaments (e.g. the comb, a fleshy red ornament on the head), and other morphological features (like body size) contribute to assessment of an antagonist, but do not fully predict the social status of an individual [25]–[27]. To assess the extent to which behavioural variation is related to the establishment of social status, we investigated if variation in behavioural responses scored in a novel arena test and a startle test could predict the outcome of duels of morphologically matched male fowl. Our expectation is that more proactive males have higher chances of becoming dominant.

## Methods

### Study population and animal housing

The study took place during the breeding seasons (June–August) of 2012 and 2013 at Tovetorp Research station, Stockholm University, Sweden. We used a chicken population of the breed ‘Swedish bantam’ (*Gallus gallus domesticus*) that has been kept at the research station since the late 60′s. This breed is behaviourally and morphologically similar to their wild ancestor, the red junglefowl (*Gallus gallus*) [28]. The chickens were kept under semi-natural conditions in six mixed-sex, mixed-age (1–10 years) groups of 15–20 individuals, and with permission from the Swedish board of agriculture. All chickens were used to human handling. We used 50 males marked with numbered leg rings to facilitate identification. In order to standardise the social position of experimental males, single males were housed together with one female in outdoor aviaries (3×3 m) for 4–5 days before the first behavioural test. Aviaries were visually, but not vocally, isolated from other birds. In this set-up, all males behaved like dominant birds prior to observations [29].

The study was conducted according to ethical requirements in Sweden. Linköping Ethical Committee reviewed and approved the study (Linköping Ethical Committee ethical permit number 60–10).

### Personality assays

All males were tested singly in a novel arena test to investigate inter-individual variation in activity, explorative behaviour, territorial crowing and vigilance. The arena was an oval shaped fenced area (11.5×10 m) in a deciduous forest, divided into 32 roughly even-sized subareas (Figure S1). The male was initially placed in a start cage of chicken wire (60×50×50 cm) connected to the arena. The door to the arena was immediately opened, after which the behaviour of the male was recorded for 20 min. All males entered the arena voluntarily within a few seconds, except for four males that entered the arena within 1–8 min. The observer sat outside the arena, visible to the male. The occurrence of ‘Activity’ (scratching on the ground, or walking) was recorded every 15 s. Exploration propensity was measured both as the number of subareas visited at least once by a male (‘Number of subareas explored’, between 1–32), and as the latency to visit five subareas (‘Latency to explore 5 subareas’). This measure was used because a majority of the males visited at least five subareas, resulting in a continuously distributed variable. The number of territorial crows uttered (‘Number of crows’), and the proportion of time a male spent being vigilant (‘General vigilance’, head above shoulder height, recorded every 15 s), were recorded. Immediately after the novel arena test, a startle test was performed by presenting a play-back of a 3 s long warning call of a conspecific using an iPhone connected to a portable loudspeaker (Philips Mighty Mini). The warning call used was a loud cackling vocalisation uttered in response to terrestrial predators, to which flock mates typically react to with increased vigilance [30]. The latency until the male resumed feeding after the startle was recorded (‘Vigilance after startle’). Males that did not resume feeding within 10 min received the maximum score of 600 s. Male behaviour was only measured once in the current study, but behavioural responses in novel arena tests (including variation in activity, exploration, and vigilance) have previously been shown to be consistent within male fowl of this population [31]. The recorded responses obtained from these tests were therefore used to describe the personality of males.

### Aggression and social status

Dyadic interactions were performed 4–8 hours after the novel arena and startle test were carried out. Duels were filmed using a digital camcorder (Sony DCR-VX1000E). Pairs of males (*n_pairs_* = 25) were simultaneously placed 1 m from each other in an outdoor aviary (2×3 m) where none of the males had recently been housed (>1 year). The males of each pair were matched for comb size and body size (<10% difference), estimated by comb length (in mm), tarsus length (in mm) and body weight (in g). Males within a pair had not been housed together for at least 2 weeks prior to duels, reducing any effects earlier encounters may have had on the establishment of social status [33]. Aggression is often positively related to the outcome of agonistic interactions e.g. [25]. To study whether behavioural responses in the personality assays were related to aggression, we scored the males’ aggression during the initial part of the duel. The intensity of fowl fights ranges from aggressive displays (raised hackles, dropped wing and ‘waltzing’ movements) to repeated attacks with the feet or the beak [34]–[35]. The males’ initial level of aggression was classified along a scale from 0–3 as either 0: ‘fleeing’ (away from the other male), 1: ‘neutral’ (straight body posture, no avoidance or attraction to the other male), 2: ‘aggressive’ (crouched body posture and dropped wing or raised hackles [36]), or 3: ‘very aggressive’ (approaching the other male within 2 s). All duels ended with one of the males avoiding the other and no longer retaliating to further attacks from the opponent. We used a minimum of five occasions of avoidance by the same male to define that male as subordinate, and the other male as dominant. The observation was terminated when this criteria was fulfilled, which could take up to one hour. Chickens that live in mix-sexed groups typically establish dominance ranks through displays and threats, thus using a minimum of physical fighting. During this study, physical fighting during dyadic interactions between males was very brief (mean 22 s±8 CI, max 153 s) and no injuries occurred other than occasional minor bleedings from the comb. These very small injuries did not require veterinary care, and did not affect the general health state of the animals. However, the research station has access to a veterinary in case animals get injured or sick. No animals were sacrificed during the study, and after the experiment, all males were returned to their regular groups in the population.

Since initial aggression and the outcome of the duel were measured in the same duel, we did not use initial aggression as a predictor of the outcome of the duel. However, aggression of a subset of the males (*n* = 31) was scored again one year later (during the breeding season of 2013) for another study. We here use this partly overlapping dataset to investigate if initial aggression during the duel may reflect individual aggression as a personality trait. In the other study, we used a novel experimental setup where aggression could be scored independently of the behaviour of the opponent. Aggression scores were estimated as the level of aggression towards a male intruder who was manually restrained in the hands of the observer, starting at 1 m distance from the focal male, in the focal male’s home cage. The presentation was terminated after 1 min, or when the focal male was about to attack, thus avoiding physical attacks on the restrained male. The males’ level of aggression was classified along a scale from 0–6 (table 1) [34], [35], [36]. Six different males were used as presented intruders, and these were chosen to have approximately equal or smaller comb compared to the focal male (mean difference between focal and presented male −10.3% ±11.5 (SD)). The Swedish bantam is a small breed where males rarely exceed 1.4 kilo in bodyweight. In other words, males are rather small and when presented by being held in the hands of a person, most of the body is covered. The sight of the neck and head of the presented male, and some of the tail and legs is enough to trigger an aggressive response in the focal male. Presenting males in a standardised position held by the observer most likely reduce the assessment of the body size of the presented male by the focal male. We therefore did not match males with regards to body size.

**Table 1:** Definition of aggression scores of male fowl in response to the presentation of a manually presented intruder.

Score	Description
0	Walks away from the other male
1	Straight body posture, no avoidance or attraction to the other male
2	Standing still, dropped wing [1]
3	Aggressive posture [1], [2]
4	Aggressive posture + aggressive ground pecking [3] within 30 sand/or approach opponent <50 cm within 60 s
5	Aggressive posture + approach opponent <50 cm within 30 s
6	Approach opponent <20 cm within 10 s

The scores range from 0 to 6 (6 being highest). Males with a crouched body posture and dropped wing were considered to have a fully aggressive body posture (score 3). Males that in addition performed the aggressive display ground pecking, or approached or attacked the presented male, received a higher score.

### Statistical analyses

The relationships between behavioural responses in the novel arena test (Activity, Number of subareas explored, Latency to explore 5 subareas, Number of crows, General vigilance, Vigilance after startle) and initial aggression during the duel were analysed by Spearman rank correlations.

Comparisons of behavioural responses in the novel arena test and the startle test between males that later became dominant and subordinate were performed by Wilcoxon matched pairs test (*n_pairs_* = 25). Because of strong correlations between the responses related to activity and exploration, these responses were merged to one single variable (see below).

The relationship between initial aggression and the outcome of the duel was analysed by comparing the aggression scores for the winner and the loser of a pair by a Wilcoxon matched pairs test. A linear mixed model was used to compare aggression scores obtained during the initial part of the duel and towards a manually presented intruder (*n* = 31). Aggression towards the presented intruder was used as response variable and the initial aggression during the duel as explanatory variable. Individual outcome of the duel (dominant or subdominant) was used as a fixed-effect factor. The duel pair identity, and the identity of the presented intruder were used as random-effect factors in order to control for their potential influence on aggression scores of individuals.

R version 3.0.3 was used for all statistical analyses, using the package lme4 version 1.1–5 [36], [37].

## Results

The three measures of activity/exploration (Activity, Number of subareas explored and Latency to 5 subareas) were strongly correlated (all r>0.70, all *p*<0.001). These responses were therefore merged by calculating a mean for each male after standardising the variables by dividing them with their standard deviation. Latency to explore 5 subareas was first multiplied by −1 to obtain a variable where a higher value described males that were more explorative. The merged activity/exploration measure was used in all further analyses, and is called ‘Exploration’ throughout. After the merging of these variables, the relationship between the behavioural responses obtained from the novel arena and the startle test (Exploration, Number of crows, General vigilance and Vigilance after startle) and initial aggression during the duel were analysed by Spearman rank correlations (table 2). There were no strong correlations neither among the behavioural responses of the novel arena test, nor between aggression at the beginning of the duel and any of the other behavioural responses recorded (table 2). There was a moderate positive correlation between General vigilance and Vigilance after startle (table 2).

**Table 2:** Correlations among behavioural responses of male fowl in a novel arena test, startle test and aggression score obtained during the initial part of a duel.

	Exploration	Number of crows	General vigilance	Vigilance after startle
**Exploration**	-			
**Number of crows**	0.13	-		
**General vigilance**	0.26	0.20	-	
**Vigilance after startle**	0.021	0.06	0.28*	-
**Aggression score in duel**	0.068	–0.21	0.15	0.04

Spearman rank correlation coefficients (r) are shown.

*denotes significance at the *p*<0.05 level.

**Figure 1:**
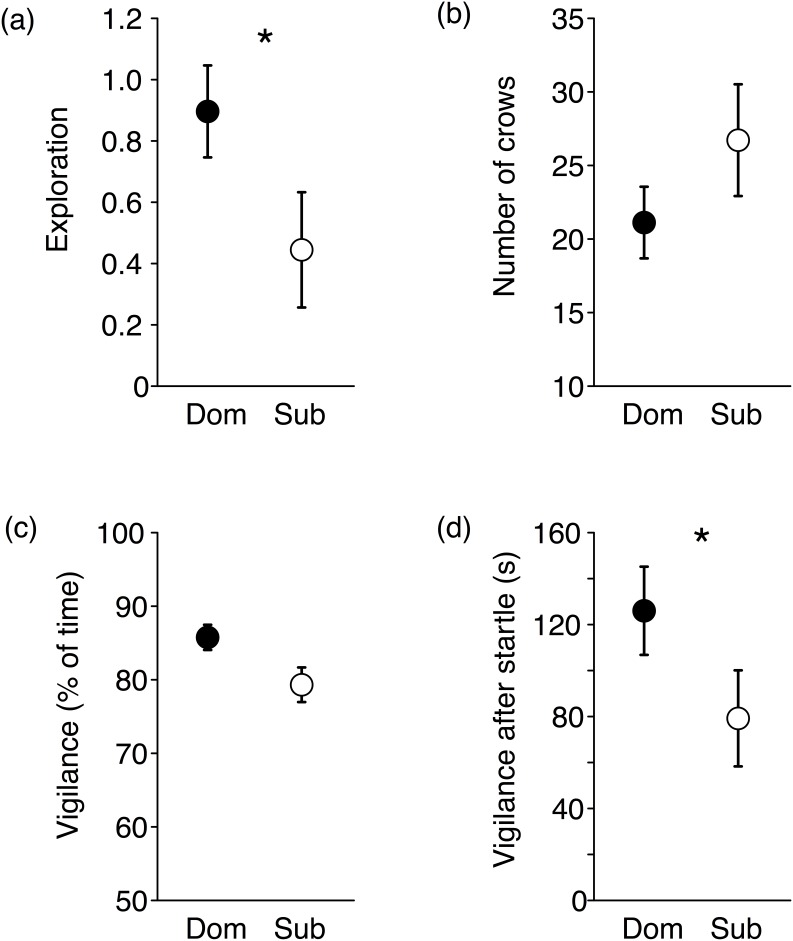
Behavioural responses in behavioural assays of future dominant and subordinate male fowl. Males that later became dominant (filled dots) were (a) more explorative compared to males that later became subordinate (open dots, Exploration score is presented as SD units, see text for further explanation). There was no difference in (b) the number of crows uttered or (c) general vigilance in the novel arena test between future dominant and subdominant males. Future dominant males (d) remained vigilant for a longer period of time after a startle (a playback of conspecific warning call). Mean values ± SE are given. *p<0.05.

Males that later became dominant were more explorative than future subordinate males (z  = −2.38, p = 0. 017, Figure 1a). There was no difference between future dominant and subordinate males in the number of crows uttered (z  = −1, p = 0.32, Figure 1b), or general vigilance in the novel arena test (z  = −1.55, p = 0.12, Figure 1c). Vigilance after playback of a warning call was predictive of future social status. Future dominant males were vigilant for a longer period post startle compared to males that later became subordinate (z  = −2.10, p = 0.036, Figure 1d).

**Figure 2:**
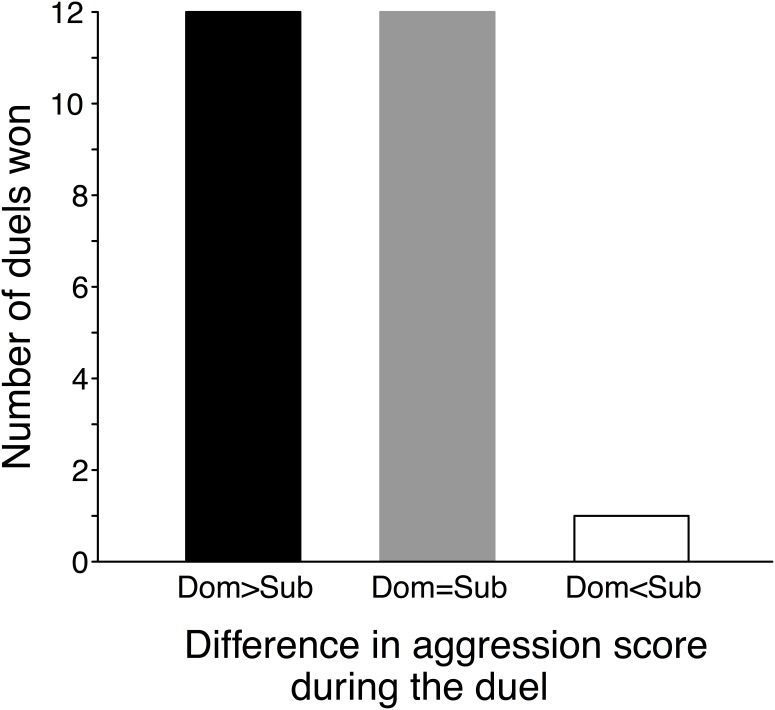
The distribution of differences in aggression score of male fowl in duels. The winning, dominant male either had a higher aggression score compared to the losing, subordinate male (Dom>Sub, black column), the same aggression score (Dom = Sub, grey column) or a lower score (Dom<Sub, white column). When aggression scores differed between the two males of a pair, showing higher levels of aggression during the initial part of the duel was associated with winning it (12 cases vs 1 case).

The winning male started the duel with a more aggressive body posture compared to the losing male (mean_dom_ = 2.16±0.13 CI, mean_sub_ = 1.6±0.13 CI, z  = −2.47, p = 0.013). Paired males differed in aggression score at the beginning of the duel in 13 out of 25 duels, and in 12 out of these, the male with the higher score won the duel (Figure 2). Initial aggression during the duel had a significant positive relationship with aggression towards a manually presented intruder one year later (χ^2^ = 6.9, df = 1, p = 0.0085). All other factors included in the model were insignificant (see table 3 for further details).

**Table 3:** Linear mixed-model analysis of aggression scores shown by male fowl towards a manually presented intruder.

Fixed effect	Estimate	SE	p
Aggression score in duel	1.61	0.61	0.0085
Duel outcome	0.99	0.74	0.18
**Random effect**	**Variance**	**Std.Dev**	
Duel pair	0	0	
Id presented intruder	0	0	
Residual	3.13	1.77	

The aggression score (0 to 6) of a male towards a restrained intruder was used as response variable, and the aggression score (0 to 3) during the initial part of the duel was used as an explanatory variable, with the individual outcome of the duel as a fixed-effect factor (*n* = 31). Aggression towards a restrained intruder had a significant positive relationship with aggression during the duel.

## Discussion

Socially dominant individuals typically have increased access to resources, in turn resulting in improved reproductive success. The social position of a group-living individual can therefore be crucial for an individual’s fitness, and group members commonly compete to become dominant [2]. Here, we demonstrate that variation in personality of male fowl affected the chances of obtaining a high social status for individuals with size-matched morphological traits.

Dominant male fowl differ quantitatively from subordinate males in their behaviour, for example they crow more [24], [31], [39]. Crowing is an auditory display, which signals dominance and territoriality, and dominant males often interfere with subordinate males that attempt to crow [39], [40]. Dominant males are also more vigilant both when in a social context in mixed-sex groups, and when temporarily isolated in a novel arena test [24], [31], [40]. However, we found no support for pre-existing differences between future dominant and subordinate individuals in neither how much a male crowed, nor how much time he spent being vigilant in the novel arena test. The differences in vigilance and crowing observed in other studies [24], [31], [39], [41]–[43] therefore primarily seem to be an effect of the current social position rather than pre-existing differences in behavioural type, and might be enforced by the presence of conspecifics [31], [42]–[43]. Dominant males have an increased access to females, and increased mate guarding can shift the trade-off between foraging and vigilance towards more vigilance [44]. Vigilance further plays a role in predator detection e.g. [45], but it is an open question if an increase in assets through dominance causes males to be more risk-averse (i.e. more vigilant and alert), although this would be consistent with the asset protection principle [46], [47]. We found that, when startled by the play-back of a conspecific’s warning call typically uttered when ground predators are detected, future dominant males remained vigilant for a longer period of time. This indicates that vigilance in a more risky context, here simulated by a conspecific’s detection of a potential predator, may predict social status. The moderate positive correlation between the two measures of vigilance indicates that vigilance across contexts at least in part describes the same personality trait. Further studies are required to investigate the functional significance of the observed variation in vigilance.

In line with a study with zebra finches (*Taeniopygia guttata*), fast exploring males more often became dominant [23]. This result suggests that being an active and explorative individual brings advantages to a duel situation. Our results suggest that this advantage is not necessarily through a direct link to aggression. In fact, none of the personality traits measured in the personality assays we used correlated with aggression at the start of the duel, which indicates that exploration and vigilance predicted social status independently of aggression in male fowl.

That aggression during dominance interactions is positively related to the outcome has been demonstrated in earlier studies of various species e.g. [11] including the domestic fowl, where females that initiate the first attack or have a more aggressive posture more often also win the fight [35], [36], [48]. We found that aggression at the initial part of the duel reflected the outcome of duels also between male fowl. The male that started out being more aggressive at the beginning of the duel almost always won the fight. However, in around half of the duels both males initially adopted an aggressive body posture, and thus had the same aggression score. Since aggression was scored during the same duel as the outcome was measured, there is potential circularity in the ability of this measure to predict the outcome of a duel. We therefore cannot fully disentangle the role of individual variation in aggression, in predicting social status. Further, aggression is sometimes considered a property of the interaction between individuals, in which case an individual’s aggressive response depends on the properties and actions of the opponent [49]. For instance, whether to initiate an attack or not can depend on self-assessment of the own strength, but can also depend on the estimated chances to win based on traits or actions of the opponent [50]. One year later we developed a new technique to estimate individual aggressiveness with a decreased influence of the behaviour of the opponent. Presenting a restrained male effectively eliminated potentially confounding effects of variation in opponent behaviour and elicited the same repertoire of behavioural responses as observed by focal males towards a free ranging opponent. The significant positive relationship between aggression scores obtained during the duel and when presenting a manually restrained male one year later, indicates that this behavioural trait describes consistent variation in male aggression and that it does not solely depend on the opposition. Instead, our results suggest that aggression measured at the beginning of the duel estimated the males’ internal motivation to fight, perhaps through self-assessment of their own fighting ability. Further studies are required to investigate whether aggression can predict social status when measured in a context independent of the social situation where the result is obtained.

Being more explorative is considered proactive on the proactive-reactive-continuum [12], [13], and the results are therefore in congruence with studies of other species, which show a general pattern of more proactive individuals having higher chances of becoming dominant [10], [11], [22], [23], [51]. However, there were no strong correlations between behavioural responses observed in the novel arena test, when exposed to a startle and during duels, other than between activity and the two measures of exploration. In other words, we found no support for an overall behavioural syndrome categorising individuals along a proactive-reactive continuum, which is sometimes found in other species [12].

Overall, our results demonstrate that when combatants were matched for morphological characters, behavioural traits do play an important role in the establishment of social status. We identified exploration and vigilance in a potentially risky situation as the prior attributes, or personality traits, useful for prediction of a dominant social position. Our results also demonstrate that variation in several independent personality gradients can influence the ability of an individual to obtain a higher status. To better understand the relationship between personality and dominance, the exact pathway through which exploration, vigilance and aggression affect the outcome of duels needs further investigation, in particular when aggression and other personality traits do not correlate.

### Data Accessibility

Data deposited in the Dryad Repository (http://datadryad.org/): http://doi:10.5061/dryad.885g3.

## Supporting Information

Figure S1 **Schematic drawing of the novel arena used to score variation in behaviour of male fowl.** The arena was constructed by fencing an oval shaped area in a deciduous forest. The arena was divided in 32 approximately same-sized subareas, marked by wooden sticks. A start cage made of chicken wire was attached to the arena and is denoted “S” in the figure.
(TIFF)
