# Supplementary figures and images for: Personality Predicts Social Dominance in Male Domestic Fowl
Source: PLoS One. 2014 Jul 29;9(7):e103535. doi: 10.1371/journal.pone.0103535 (PMC4114777; doi:10.1371/journal.pone.0103535)

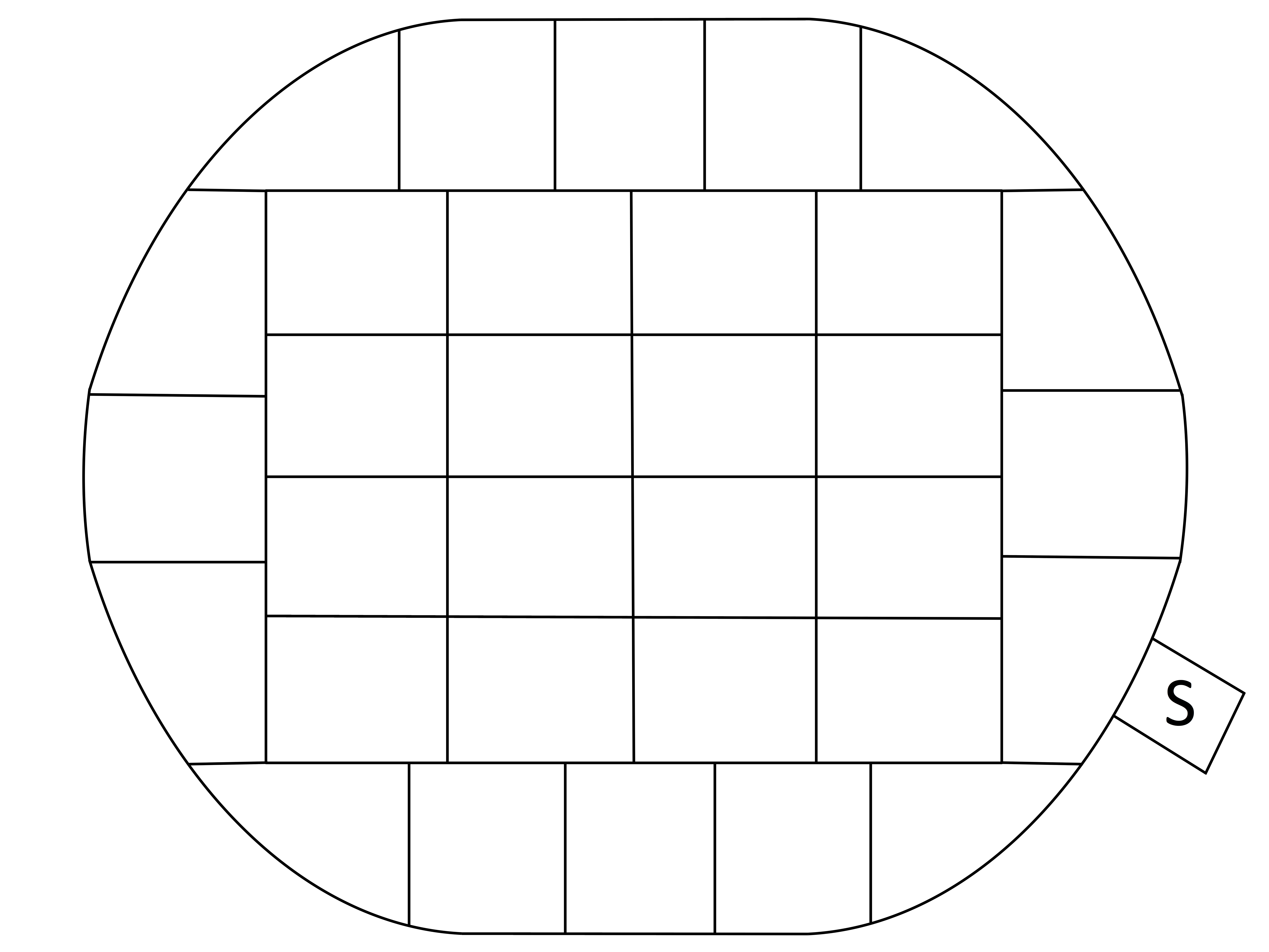

Supplement: Figure S1 — Schematic drawing of the novel arena used to score variation in behaviour of male fowl. The arena was constructed by fencing an oval shaped area in a deciduous forest. The arena was divided in 32 approximately same-sized subareas, marked by wooden sticks. A start cage made of chicken wire was attached to the arena and is denoted “S” in the figure. (TIFF) [file pone.0103535.s001.tif]
